# Harmonic Allocation of Authorship Credit: Source-Level Correction of Bibliometric Bias Assures Accurate Publication and Citation Analysis

**DOI:** 10.1371/journal.pone.0004021

**Published:** 2008-12-24

**Authors:** Nils T. Hagen

**Affiliations:** Faculty of Biosciences and Aquaculture, Bodø University College, Bodø, Norway; American Museum of Natural History, United States of America

## Abstract

Authorship credit for multi-authored scientific publications is routinely allocated either by issuing full publication credit repeatedly to all coauthors, or by dividing one credit equally among all coauthors. The ensuing inflationary and equalizing biases distort derived bibliometric measures of merit by systematically benefiting secondary authors at the expense of primary authors. Here I show how harmonic counting, which allocates credit according to authorship rank and the number of coauthors, provides simultaneous source-level correction for both biases as well as accommodating further decoding of byline information. I also demonstrate large and erratic effects of counting bias on the original *h*-index, and show how the harmonic version of the *h*-index provides unbiased bibliometric ranking of scientific merit while retaining the original's essential simplicity, transparency and intended fairness. Harmonic decoding of byline information resolves the conundrum of authorship credit allocation by providing a simple recipe for source-level correction of inflationary and equalizing bias. Harmonic counting could also offer unrivalled accuracy in automated assessments of scientific productivity, impact and achievement.

## Introduction

Modern science is dominated by multi-authored publications [1], yet there is no consensus on how to allocate authorship credit for multi-authored papers [2]. Nevertheless, authorship credit is routinely allocated either by issuing full publication credit repeatedly to all coauthors, or by dividing one credit equally among all coauthors [3]. The ensuing inflationary and equalizing biases have the capacity to distort bibliometric indices, confound research evaluation [cf. 3], [4]–[6], and systematically benefit secondary authors at the expense of primary authors.

Correcting for the equalizing bias inherent in both allocation schemes requires either a total reliance on explicit contribution statements [7], or a detailed decoding of existing byline information to ensure accurate allocation of publication and citation credit according to authorship rank and other relevant information. The latter is still unresolved after 40 years of debate [1]. The former solution, although recommended as a remedy for “honorary authorship” and other inappropriations [8], is still far from being universally adopted. Meanwhile there is controversy over the validity of judging science by equating merit with publishing performance [12]–[16], and the future direction of science is being influenced by hiring committees, funding agencies and officials using biased and incompletely tested bibliometric measures [9]–[11].

Here, I identify equalizing and inflationary counting bias as the two main varieties of bibliometric bias and show how a popular bibliometric measure, the *h*-index [17], is distorted by these biases. I advocate the use of a novel harmonic counting scheme that simultaneously corrects both biases by allocating publication and citation credit according to authorship rank and the number of coauthors. I also show how harmonic counting accommodates further decoding of byline information. Finally, I emphasize the decisive importance of source-level bias correction for the outcome of automated ranking procedures, and conclude that harmonic counting provides a transparent protocol for critically enhancing the accuracy and credibility of bibliometric research evaluation.

## Results and Discussion

### Harmonic Counting Corrects Bibliometric Bias

**Figure 1:**
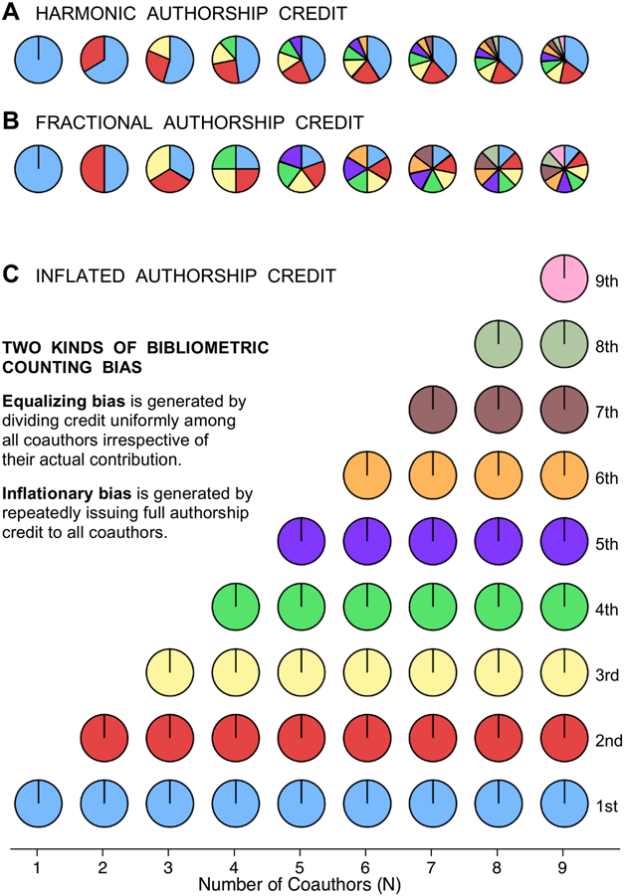
The authorship pie. (A) Unbiased harmonic allocation of publication credit according to authorship rank and the number of coauthors. (B) Fractional allocation of equal credit to each coauthor generates equalizing bias. (C) Inflated allocation, whereby full publication credit is issued repeatedly to all coauthors, generates equalizing and inflationary bias. Ordinal numbers indicate color coding for authorship rank.

Current measures of scientific publication performance routinely rely on two counting methods: inflated counting (Figure 1C), where full authorship credit is issued repeatedly to all coauthors (also known as total, normal, or standard counting), and fractional counting, where one credit is divided equally among all coauthors (Figure 1B, 2B) [3], [18]. Fractional counting corrects for **inflationary bias** generated by the multiple counting of multi-authored publications (Figure 1C) [cf. 6], but both counting methods generate **equalizing bias** by dividing credit uniformly among all coauthors, irrespective of their actual contribution (Figure 1B, 1C).

**Figure 2:**
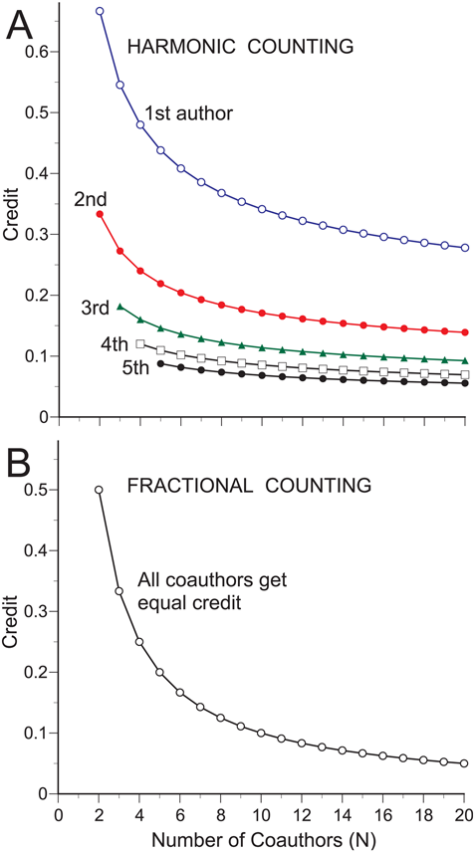
Counting credit for multi-authored publications. (A) Harmonic allocation of authorship credit. (B) Fractional allocation of authorship credit.

Harmonic counting of publication credit, although not named as such, was proposed in 1981 by Hodge and Greenberg [19], in response to a plea for fractional allocation of publication credit by Price [20], but to the best of my knowledge has never been implemented in a bibliometric context. Harmonic counting simultaneously removes both inflationary and equalizing bias by allocating publication and citation credit according to authorship rank and the number of coauthors (Figure 1A, 2A).

The harmonic credit for the *i* ^th^ author of a publication with *N* coauthors is calculated as follows:
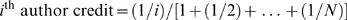
This formula ensures that:

1. total publication credit is shared among all coauthors,
2. the first author gets the most credit, and in general the *i* ^th^ author receives more credit than the (*i*+1)^th^ author, and
3. the greater the number of authors, the less credit per author.

**Figure 3:**
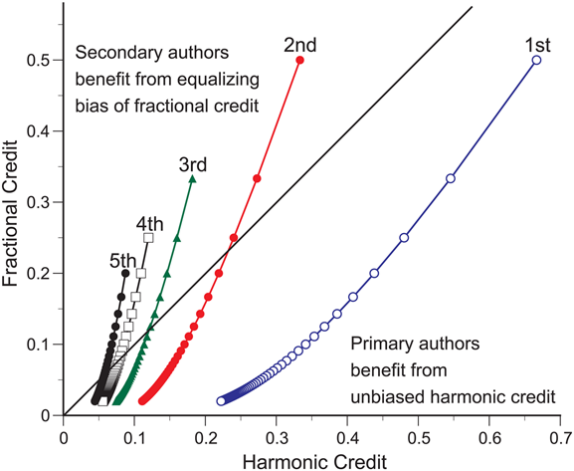
The plight of primary authors. Curves comparing harmonic and fractional allocation of publication or citation credit are plotted for the first 5 authors for publications with up to 50 coauthors. Points above the diagonal line indicate authors benefiting from the equalizing bias of fractional counting. Curves crossing the diagonal line indicate how authors lose the initial benefit of fractional counting as the number of coauthors increases, i.e. secondary authors become primary authors when they no longer benefit from equalizing bias.

In contrast, biased counting systematically benefits secondary authors at the expense of the primary authors who, in the absence of byline information to the contrary, presumably earned their authorship rank by contributing more. Primary authors are located in the lower half of Figure 3, and the transition between secondary and primary authorship is illustrated by curves crossing the diagonal line. First authors are always classified as primary authors. Subsequent authors are initially classified as secondary authors but lose the initial benefit of fractional counting when the number of coauthors increases, and become primary authors when they no longer benefit from equalizing bias.

### Harmonic *h*-index

Bibliometric counting bias affects all derived measures of per capita scientific production, impact and achievement, including publication metrics, citation metrics, and the *h*-index—a heuristic metric of merit that combines inflated counts of publication and citation data for an individual author into a single integer *h*, equal to the number of publications with at least *h* citations [17].

To illustrate the erratic effects of counting bias on the *h*-index, I compared *h*-index scores calculated from harmonic, fractional, and inflated non-self citation counts [21], for an anonymized sample of 11 associate professors and 9 full professors at the Faculty of Biosciences and Aquaculture (FBA), Bodø Regional University, Norway (Table 1).

**Table 1:** Effect of counting bias on the *h*-index scores of associate (Aspro) and full (Pro) professors at the Faculty of Biosciencess and Aquaculture, Bodø, Norway.

Staff	*h*-index scores by counting method (Relative subgroup rank)			Counting bias	
	Harmonic	Fractional	Inflated	Equalizing	Inflationary
Aspro09	6 (1st)	5 (1st)	10 (1st)	−1	5
Aspro06	5 (2nd)	4 (2nd)	5 (5th)	−1	1
Aspro02	4 (3rd)	5 (1st)	6 (4th)	1	1
Aspro03	4 (3rd)	2 (4th)	5 (5th)	−2	3
Aspro04	4 (3rd)	4 (2nd)	7 (3rd)	0	3
Aspro05	4 (3rd)	4 (2nd)	8 (2nd)	0	4
Aspro08	4 (3rd)	4 (2nd)	4 (6th)	0	0
Aspro10	3 (4th)	3 (3rd)	4 (6th)	0	1
Aspro01	2 (5th)	2 (4th)	5 (5th)	0	3
Aspro07	1 (6th)	0 (5th)	2 (7th)	−1	2
Aspro11	0 (7th)	0 (5th)	2 (7th)	0	2
Pro09	10 (1st)	10 (1st)	20 (1st)	0	10
Pro03	8 (2nd)	8 (2nd)	11 (2nd)	0	3
Pro05	6 (3rd)	6 (3rd)	10 (3rd)	0	4
Pro06	6 (3rd)	4 (5th)	9 (4th)	−2	5
Pro07	6 (3rd)	6 (3rd)	6 (6th)	0	0
Pro04	5 (4th)	5 (4th)	11 (2nd)	0	6
Pro08	5 (4th)	5 (4th)	5 (7th)	0	0
Pro01	4 (5th)	2 (6th)	7 (5th)	−2	5
Pro02	1 (6th)	1 (7th)	2 (8th)	0	1

Harmonic scores are correct for both equalizing and inflationary bias, fractional scores are corrected for inflationary bias, and inflated scores are corrected for neither. All scores are corrected for self-citation.

**Figure 4:**
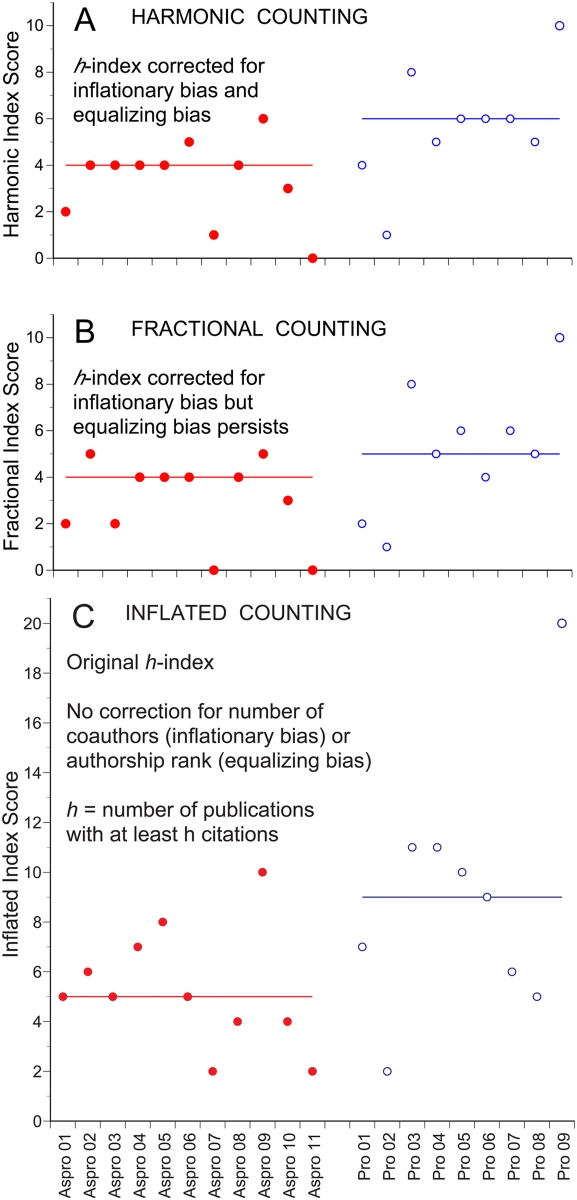
The impact of bibliometric counting bias on *h*-index scores. (A to C) Comparing the effects of inflationary and equalizing bias on individual *h*-index scores and subgroup median scores (horizontal lines) for a sample of associate professors (Aspro, filled symbols) and full professors (Pro, open symbols) from the Faculty of Biosciences and Aquaculture (FBA), Bodø Regional University, Norway. The sample includes all publications by 20 FBA staff members listed in the ISI and SCOPUS databases in December 2007, i.e. a total of 364 publications, in 124 journals, with 3685 non-self citations.

The *h*-index scores of 80% of the FBA staff were altered by the combined effect of unidirectional inflationary bias and bidirectional equalizing bias, i.e. by the difference between harmonic and inflated *h*-index scores (Figure 4, A and C, Table 1). This in turn altered the within subgroup rankings of a different 80% subset of the staff, the end result being that all members of the FBA staff were affected by counting bias.

Contrasting harmonic and fractional *h*-index scores shows how bidirectional equalizing bias altered the scores of 35% and distorted the within subgroup rankings of 60% of the FBA staff (Figure 4, A and B, Table 1). The negative net effect of equalizing bias (1 positive, 6 negative) indicates a preponderance of adversely affected primary authors in the sample.

Unidirectional inflationary bias, i.e. the difference between fractional and inflated scores, increased the *h*-index of 85% of the FBA staff and distorted the within subgroup rankings of 70% of the staff members (Figure 4, B and C, Table 1). Inflationary bias doubled the maximum *h*-index score (from 10 to 20), and distorted mid level scores erratically, e.g. a fractional *h*-index score of 4 corresponded to inflated scores ranging from 4 to 9. Two recent publications evaluating the effect of fractional counting on the *h*-index similarly found that fractional index scores were reduced to 58–86% of their original values [22], [23].

Comparing the range of overlap in *h*-index scores between associate professors and full professors shows that the harmonic *h*-index minimized the range of overlap to a level where it would have been eliminated entirely by promoting the two highest ranking associate professors and demoting the two lowest ranking full professors (20% of the staff, Figure 4A). This result indicates that the harmonic *h*-index might also find application as an impartial indicator of premature or overdue promotions.

Hirsch's [24] suggestion that using the original *h*-index “… *as a measure of scientific achievement automatically reduces an important source of distortion when multiply coauthored papers are involved, by allocating a smaller portion of the credit to those authors who are likely to have contributed less.*” is not supported by my results. Hirsch correctly identified uniform allocation of authorship credit as a source of distortion, but since this distortion is a direct result of using biased counting when calculating the *h*-index it must be remedied by removing such bias prior to calculation, i.e. by using harmonic counting to remove both inflationary and equalizing bias from the source data. The resulting harmonic *h*-index meets the expressed intention of Hirsch by automatically allocating citation credit according to the relative contribution of each coauthor, while retaining the essential simplicity, transparency and intended fairness of the original *h*-index [17]. The harmonic *h*-index would therefore appear to be a superior choice for bibliometric ranking of individual scientific merit.

### Further Decoding of Byline Information

Harmonic counting corrects inflationary and equalizing bias by decoding byline information on the assumption that the authorship rank indicated in the byline hierarchy accurately reflects the actual magnitude of each coauthor's contribution. This assumption appears to be valid for the present sample, as I was unable to detect any evidence of alphabetical or randomized ranking.

**Figure 5:**
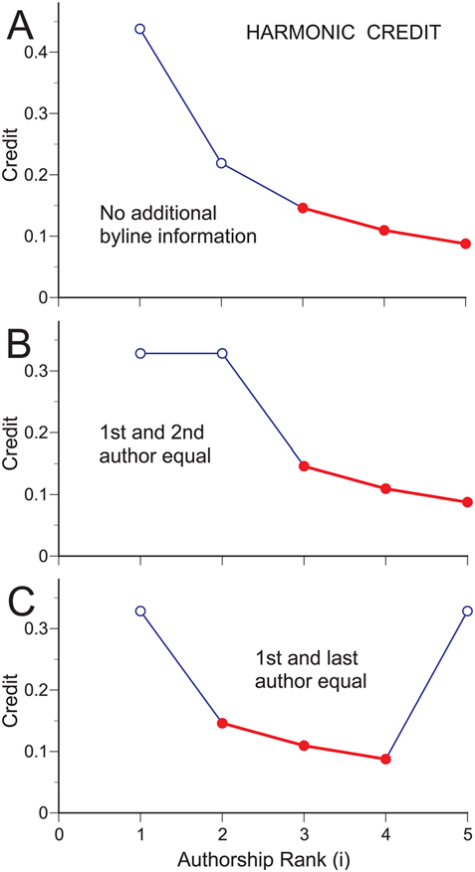
Harmonic bibliometric credit allocation according to authorship rank and byline information. (A) Harmonic counting allocates credit as a decreasing function of authorship rank when additional byline information is non-existent or disregarded. (B) Allocating equal credit to adjacent coauthors does not alter the amount of credit allocated to the remaining coauthors. (C) Allocating equal credit to non-adjacent coauthors reduces the credit of intermediate coauthors by one position. This is accomplished by promoting the lower ranking equal until the equals are adjacent. For example, when the first and last authors are equal, the credit allocated to the last author is equivalent to that of an equal 2^nd^ author (as depicted in panel B). Primary authors (open symbols), and secondary authors (filled symbols) are plotted for a paper with 5 coauthors.

Nevertheless, additional byline information may provide explicit instruction about the equality of some coauthors' contributions, or implicit information about the approximate equality of contributions by first and last authors, as in biomedical research where the corresponding author is customarily listed last [25], [26]. Such variations are easily accommodated by a harmonic counting scheme with little or no alteration of the credit allocated to the remaining coauthors (Figure 5, A to C). To wit, allocating equal credit to adjacent coauthors does not alter the amount of credit allocated to the remaining coauthors (Figure 5B), and allocating equal credit to non-adjacent coauthors simply demotes intermediate authors by one position. This is accomplished by promoting the lower ranking equal until the equals are adjacent. For example, when the first and last authors are equal (Figure 5C), the credit allocated to the last author is equivalent to that of an equal 2^nd^ author (Figure 5B), thereby reducing the credit of intermediate coauthors by one position. This simple scheme indicates that harmonic credit thus allocated offers a level of bibliometric accuracy that can only be surpassed by the universal adoption of explicit contribution statements.

**In conclusion,** I emphasize the decisive importance of source-level bias correction in bibliometric research evaluation, and suggest that the harmonic *h*-index provides a transparent measure of scientific merit that would critically enhance the accuracy and credibility of automated ranking procedures. In turn, removing bias from bibliometric research performance measures would facilitate independent post hoc analysis of the differences between peer judgement and automated ranking of merit. Such bias removal might also provide the impetus for reappraisal of field-specific differences in publishing behavior and suggest alternative explanations for the explosive increase in the number of coauthors. To enable source-level correction of inflationary and equalizing biases would require implementation of easily accessible options, similar to the options for source-level correction of self-citation bias recently added to the ISI and SCOPUS databases.

## Materials and Methods

The dataset consists of the publication and citation records of 20 scientists, 11 associate professors and 9 full professors, currently working at the Faculty of Biosciences and Aquaculture (FBA), Bodø Regional University. The collective publication output of the FBA staff consists of 364 publications in 124 journals, with a total of 3685 citations, a range of 0–152 citations per paper, and a median of 5 citations per paper (mean 10.35). Only 9 publications had more than 1 coauthor among the present staff members.

Publication and citation data were obtained from the ISI and SCOPUS databases in December 2007. Self-citations were excluded manually by checking the citation records for all publications recorded in either database. Some publications not recorded in either database were located by using the Cited Author Search feature of ISI. It was impractical to check the citation records of these publications manually, but I assessed the potential effect of self-citation in these records on individual *h*-index scores, and judged it to be inconsequential.

The publication and citation coverage of ISI and SCOPUS varied. SCOPUS listed 2315 citations from 237 publications, whereas ISI listed 3030 citations from 310 publications. I compared citation counts for the 219 publications listed in both databases (60% of the total sample), and consistently used the maximum value when citation counts differed (100 publications, 46% of subsample).
